# Ultra-processed food consumption and dietary, lifestyle and social determinants: a path analysis in Brazilian graduates (CUME project)

**DOI:** 10.1017/S1368980022002087

**Published:** 2022-12

**Authors:** Jéssica Bevenuto Mattar, Ana Luiza Gomes Domingos, Helen Hermana Miranda Hermsdorff, Leidjaira Lopes Juvanhol, Fernando Luiz Pereira de Oliveira, Adriano Marçal Pimenta, Josefina Bressan

**Affiliations:** 1 Department of Nutrition and Health, Universidade Federal de Viçosa (UFV), PH Rolfs Avenue W/N, University Campus, Viçosa, MG 36571-000, Brazil; 2 Department of Statistics, Universidade Federal de Ouro Preto (UFOP), Ouro Preto, MG, Brazil; 3 Department of Nursing, Universidade Federal do Paraná (UFPR), Curitiba, PR, Brazil

**Keywords:** Eating behaviour, Lifestyle, Nutritional epidemiology, Industrialised foods, NOVA classification

## Abstract

**Objective:**

To explore the relationship between ultra-processed foods (UPF) consumption and dietary, lifestyle and social determinants using pathway analysis in the baseline of the Cohort of Universities of Minas Gerais (CUME project).

**Design:**

Cross-sectional study, in which path analysis was used to estimate direct and indirect effects of dietary practices, sleep, time on the computer and professional status on UPF consumption.

**Setting:**

Data were collected in 2016, through an online questionnaire composed of sociodemographic, anthropometric, lifestyle and dietary practices questions, and a FFQ.

**Participants:**

Baseline participants from the CUME Project (*n* 2826), adults who graduated from Universidade Federal de Viçosa or Universidade Federal de Minas Gerais, Brazil.

**Results:**

Being employed (*P* = 0·024), the time spent on the computer (*P* = 0·031) and the frequency of fried food intake (*P* < 0·001) were positively and directly associated with UPF consumption, whereas the sleep duration (*P* = 0·007) and the number of meals per d (*P* < 0·001) were negatively and directly associated with UPF consumption. Indirect effects were observed between being employed, mediated by the sleep duration (*P* = 0·032) and fried food intake (*P* = 0·005), whereas being a student is mediated by the time on the computer (*P* = 0·048).

**Conclusion:**

The time spent on the computer, sleep duration and fried food consumption showed direct effects on UPF consumption. They also acted as mediators on the relationship between professional status and UPF consumption. Besides, the number of meals eaten each day also was directly associated with UPF consumption.

The increasing prevalence of non-communicable diseases (NCD) complicates the state of public health in Brazil^(1)^. In 2015, NCD accounted for 75·8 % of all deaths in Brazil^(2)^. This data represents a substantial reduction in quality of life, high social costs and government expenditure on health^(1)^.

Nutrition is considered as the main modifiable determinant of NCD^(3)^. Nowadays, changes in eating patterns, particularly the substitution of *in natura* and unprocessed foods for ultra-processed foods (UPF), have been reported in developed and developing countries^(1,4)^.

UPF is a term that originated from the NOVA classification, a new food classification system that categorises foods regarding the extent and purpose of their processing^(5)^. It represents industrial products ready or semi-ready for consumption, formulated from ingredients and processes of exclusively industrial use^(5,6)^.

Several properties related to UPF are problematic. They are produced from chemical additives with little or no fresh food. The primary purpose of ultra-processing is to create ready-to-eat industrial products capable of replacing naturally ready-to-eat foods (unprocessed or minimally processed), since the additives make these products look (smell, taste and texture) like real food^(5,7)^. Furthermore, due to the addition of synthetic vitamins and minerals, they are sold with health claims and thus, viewed as healthy, even though they remain unhealthy^(8,9)^. In addition, they are formulated to be habit-forming, are available at affordable prices and can be consumed anywhere and anytime, making it easier their substitute natural and minimally processed foods^(7)^.

Several studies have shown an association between the consumption of UPF and poorer diet quality^(10,11)^ and negative health outcomes worldwide^(12,13)^.

Although it is already known that a diet based on UPF increases the risk of developing NCD^(1,14,15)^, adopting a healthy diet is not merely an individual choice^(4,16,17)^, and food supply has responded to market stimuli^(18)^.

Brazil has experienced an increase in the population’s purchasing power, greater access to information, an increase in education level and a change in families’ structure, with the most significant participation of women in the labour market^(19)^. These factors stimulate an accelerated pace of life that modify perceptions, preferences and food choices^(18)^, once convenience foods are better valued for money since there is a lack of time for food preparation^(20)^.

Few studies have focused on elucidating factors that shape food decisions concerning UPF consumption^(21–24)^, and none of them with Brazilians. Besides, the UPF consumption determinants are analysed independently, although we believe these factors interact with each other. Therefore, understanding how dietary, lifestyle and social factors interrelate to determine UPF consumption through appropriate statistical methods is still necessary.

Accordingly, we hypothesised that an accelerated pace of life due to the professional situation would be related to sleep and time on the computer. Both would predict eating habits and have, as a consequence, a higher UPF consumption. This is because working long hours has been related to reducing sleep duration^(25,26)^, which is associated with changes in appetite-regulating hormones^(25,27)^. On the other hand, UPF are convenient and can be consumed in front of the computer, saving consumers time for feeding^(1)^. Furthermore, studies show that appetite increases the desire to consume high-caloric foods, such as UPF, while eating more frequently during the day reduces hunger^(28–30)^.

Thus, this study aimed to explore the relationship between UPF consumption and dietary, lifestyle, and social determinants using pathway analysis in the baseline of the Cohort of Universities of Minas Gerais (CUME project).

## Methods

### CUME project

The CUME project is a concurrent open cohort that aims to assess the impact of the Brazilian dietary pattern and nutritional transition on NCD in adults who hold bachelor and postgraduate degrees from federal higher education institutions located in Minas Gerais state, Brazil. The project’s design, methodologies and recruitment plan are published elsewhere^(31)^.

The participants received an invitation email containing a link to the CUME project (www.projetocume.com.br). After registering, the participants answered the first phase of the questionnaire, which consisted of sociodemographic, anthropometric, lifestyle and health-related questions. The participants received the second phase of the questionnaire a week after completing the first phase. The second phase was composed of a semi-quantitative FFQ and fifteen questions on dietary habits and practices.

### Subjects and study design

This cross-sectional study used baseline participants of the CUME project. A total of 3272 adults who graduated from *Universidade Federal de Viçosa* (UFV) or *Universidade Federal de Minas Gerais* (UFMG), between 1994 and 2014, filled out the CUME baseline questionnaire, from March to August 2016. We excluded individuals who resided abroad over the past year (*n* 123) or non-Brazilians (*n* 13), individuals over 60 years of age (*n* 86), women who reported being pregnant or were pregnant in the past year (*n* 137), and individuals who consumed either <500 or ≥ 6000 kcal/d^(32,33)^ (*n* 87). Thus, the present study included 2826 individuals.

It is noteworthy this article used the Strengthening the Reporting of Observational Studies in Epidemiology (STROBE) to guide its methodology.

### Study variables

#### Ultra-processed food consumption

The consumption of UPF (% of daily caloric intake) is the study’s outcome. The food intake was determined from a semi-quantitative FFQ containing 144 food items into dairy products, meat and fish, cereals and legumes, fats and oils, fruits, vegetables and greens, beverages, and other foods. Each participant reported the frequency, and the portion size of each food ingested in the previous year.

To characterise the participants’ diet quality, according to the quartiles of consumption of UPF, we evaluated the intake of calories, carbohydrates, proteins, fats (total, monounsaturated, polyunsaturated, saturated and trans), cholesterol and fibre. Nutrient intake was calculated on software Dietpro^®^, using the Brazilian Food Composition Table and the food composition table of the US Department of Agriculture.

To assess UPF, the FFQ food items were reclassified according to the degree and intent of processing based on the NOVA classification^(5)^. Also, we calculated the calorie contribution (%) from each group (unprocessed and minimally processed foods; processed culinary or food industry ingredients; processed food; and UPF products).

A specific study validated the self-reported FFQ data with a subsample from the CUME project (*n* 146). There was a moderate agreement with the values measured directly from the 24-h recalls by telephone (overall ICC = 0·44; ICC = 0·36 for *in natura* and unprocessed foods; ICC = 0·54 for processed foods; ICC = 0·60 for UPF). To evaluate the reproducibility of self-reported FFQ, the subsample answered the FFQ questionnaire twice in 1 year. There was a good agreement between the two assessments of our participants’ dietary intake (ICC = 0·76 for overall and *in natura* and unprocessed foods; ICC = 0·82 for processed and UPF)^(34)^.

### Dietary determinants

#### Dietary practices

Participants also reported their eating habits. In this study, we included the number of meals they eat each day (continuous – assessed through the question ‘How many meals do you eat/day?’) and the frequency of fried food intake (continuous, in number of times/week).

### Lifestyle determinants

#### Sleep and time on the computer

The usual daily sleep duration and the usual daily time spent on the computer were measured by the response to the question, ‘In the past 12 months, how much time on average per d (in hours) have you spent (1) sleeping at night? and (2) using the computer?’. These variables were also treated as continuous in the model.

### Social determinants

#### Professional status

The current professional situation was inquired and categorised in the following *dummy* variables (no/yes): (1) employed; (2) student; and (3) retired or home duties or unemployed. For analytical purposes, the retired or home duties or unemployed category was used as reference.

### Theoric model

**Fig. 1:**
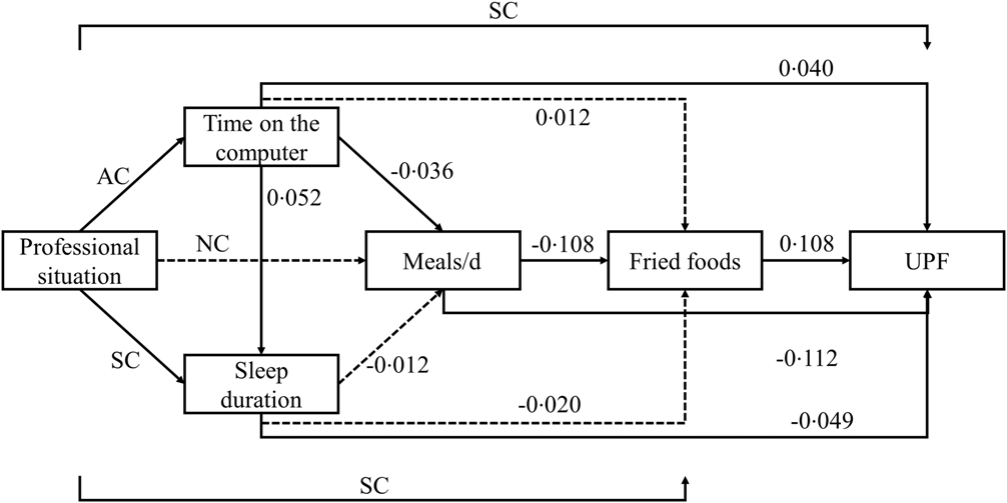
Path analysis for the relationship between UPF consumption and dietary, lifestyle, and social determinants. All variables were considered continuous, except for the professional status, which was categorised in dummy variables: employed; student; and retired or home duties or unemployed. The retired or home duties or unemployed category was used as reference. The path model was additionally adjusted by age, sex and income. Continuous lines indicate path with statistical significance (*P* ≤ 0·05). AC, all categories significants; SC, some categories significants; UPF, ultra-processed food

Figure 1 illustrates the theoretical model tested in the pathway analysis of the present study. We hypothesised that an accelerated pace of life due to the professional situation would be related to sleep and time on the computer. Both would predict eating habits and have, as consequence, a higher UPF consumption.

### Statistical analyses

To characterise the study population, absolute and relative frequencies of the socio-economic, dietary and lifestyle variables were presented for the total sample and according to UPF consumption quartiles. Trend chi-square test was used to identify statistical differences between UPF consumption quartiles. Additionally, average calories and nutrients consumed were presented as mean and standard deviation for the total sample and according to UPF consumption quartiles. One-way ANOVA and Tukey’s *post hoc* test were used for group comparisons. The descriptive analysis was performed using the SPSS^®^ software (version 23).

Path analysis was used to estimate the total, direct and indirect effects of the dietary, lifestyle, and social variables on UPF consumption. It is a multivariate statistical technique that allows performing a series of regression equations and identifying multiple relationships between variables, including mediation effects. While the direct effect concerns how much a particular independent variable (X) explains variability in a dependent variable (Y), the mediation hypotheses posit how this variable X affects the variable Y through one or more mediating variables (M). Thus, the concepts of indirect effect and total effect arise. The indirect effect of X on Y via M is defined as the product of the two coefficients linking X to Y via that mediator. The total effect of X on Y is the sum of the direct effect and all of the indirect effects^(35,36)^. In this study, the term ‘effect’ indicates a statistical association, not causality^(36)^.

The pathway analysis was performed with Mplus, version 5 (Múthen and Múthen, Los Angeles, California), using maximum likelihood estimation with robust standard errors, which does not require the assumption of normal multivariate data distribution^(36)^. Standardised coefficients (SC), expressed as units of sd, with their respective *P*-values were presented. The model was additionally adjusted by sex (categorical), age (continuous, in years) and individual income (continuous, in Brazilian currency). Model fit was assessed using multiple fit indices including the chi-square statistic, the ratio of chi-square to df (*χ* ^2^/df), comparative fit index (CFI), root mean square error of approximation (RMSEA) and standardised root mean square residual (SRMR). The sem literature suggests that model fit is excellent when chi-squares statics has no significance, *χ* ^2^/df ≤ 3, CFI ≥ 0·95, TLI ≥ 0·95, RMSEA ≤ 0·05 and SRMR ≤ 0·08^(37,38)^.

The significance level was of 5 % in all analyses.

## Results

In this study, 2826 individuals were participated, being 69·2 % female. Most of the individuals were between 30 and 39 years old (45·6 %), earned less than five minimum wages (50·3 %) and were employed (79·5 %). Individuals younger (*P* < 0·001), who earned less than five minimum wages (*P* < 0·001), who spent more than 8 h/d on the computer (*P* < 0·001) and who ate fried food at least 3 times/week (*P* = 0·002) showed a tendency of higher UPF consumption. On the other hand, individuals between 50 and 59 years old (*P* < 0·001) and who earned more than ten minimum wages (*P* < 0·001) showed lower UPF consumption (Table 1).

**Table 1:** Characteristics of the participants according to quartiles of ultra-processed food consumption, CUME project (baseline data)

	Total		1st quartile		2nd quartile		3rd quartile		4th quartile		*P*-trend
	*n*	%	*n*	%	*n*	%	*n*	%	*n*	%	
Sex (*n* 2826)											
Female	1955	69·2	483	67·7	481	67·7	489	69·8	502	71·6	0·078
Age group (*n* 2826)											
20 to 29 years	770	27·2	141	19·8	163	22·9	204	29·1	262	37·4	**< 0·001** †
30 to 39 years	1290	45·6	295	41·4	348	48·9	330	47·1	317	45·2	
40 to 49 years	525	18·6	181	25·4	140	19·7	113	16·1	91	13·0	
50 to 59 years	241	8·5	96	13·5	60	8·4	54	7·7	31	4·4	
Individual income* (*n* 2294)											
< 5 wages	1155	50·3	257	45·3	286	47·6	281	49·9	331	58·8	**< 0·001** †
≥ 5 and < 10 wages	755	32·9	190	33·5	211	35·1	193	34·3	161	28·6	
≥ 10 wages	384	16·7	120	21·2	104	17·3	89	15·8	71	12·6	
Professional status (*n* 2826)											
Employed	2248	79·5	564	79·1	584	82·1	562	80·2	538	76·7	0·919
Student	383	13·6	81	11·4	86	12·1	96	13·7	120	17·1	
Retired/unemployed	195	6·9	68	9·5	41	5·8	43	6·1	43	6·1	
Time on computer (*n* 2825)											
≤ 8 h/d	2327	82·4	618	86·7	588	82·7	573	81·9	548	78·2	**< 0·001** †
> 8 h/d	498	17·6	95	13·3	123	17·3	127	18·1	153	21·8	
Sleep duration (*n* 2825)											
≤ 6 h/night	947	33·5	227	31·8	231	32·5	234	33·4	255	36·4	0·068
> 6 h/night	1878	66·5	486	68·2	480	67·5	466	66·6	446	63·6	
Meals eaten/d (*n* 2826)											
≤ 3 meals/d	588	20·8	150	21·0	135	19·0	139	19·8	164	23·4	0·252
≥ 4 meals/d	2238	79·2	563	79·0	576	81·0	562	80·2	537	76·6	
Fried food intake (*n* 2789)											
≤ 2 times/week	1793	64·3	536	77·3	465	66·0	416	59·8	376	54·1	**0·002** †
≥ 3 times/week	996	35·7	157	22·7	240	34·0	280	40·2	319	45·9	

* Minimum wage (R$ 880·00 in 2016).

† Statistical significance (*P* < 0·05).

The average caloric consumption was 2382·3 kcal/d (± 938·1). The UPF represented 25·3 % (± 10·7) of this intake. The UPF consumption mean was 12·4 % (± 4) in the first quartile and 39·5 % (± 6·7) in the last quartile. Those participants in the first quartile of UPF consumption had a higher intake of protein, monounsaturated fat, cholesterol and fibre, while the last quartile showed higher intake of saturated and polyunsaturated fat intake. The *trans*-fat intake increased significantly across quartiles (Table 2).

**Table 2:** Nutrient intake according to quartiles of ultra-processed food consumption, CUME project (baseline data)

Daily nutrient intake	Total (*n* 2826)		1st quartile (*n* 713)		2nd quartile (*n* 711)		3rd quartile (*n* 701)		4th quartile (*n* 701)	
Total energy (kcal)	2382·3	938·1	2398	961·6^a^	2353·8	895·6^a^	2343·8	903·6^a^	2433·8	988·1^a^
Carbohydrate (g)	276·1	115·7	273	126·3^a^	278·2	112·1^a^	272·2	109·3^a^	281·1	114·1^a^
Protein (g)	107·8	54·4	113·4	63·9^a^	108·3	53^ab^	106·6	51·4^ab^	102·9	47·5^b^
Fat (g)	89·5	45·9	90·4	52·3^a^	85·1	39·5^ab^	87·2	41·4^ab^	95·4	48·6^ac^
MUFA (g)	36·8	20·6	39·9	25·9^a^	35·6	18·5^b^	35·2	18^b^	36·6	18·7^b^
PUFA (g)	16·1	9·2	15·5	9·9^a^	15·2	7·7^a^	15·8	8·7^a^	17·8	9·9^b^
Saturated fat (g)	31·5	16·4	29·8	17·3^a^	30·4	14·7^a^	31·8	15·8^a^	34·2	17·3^b^
*Trans*-fat (g)	1·1	0·8	0·8	0·6^a^	1	0·7^b^	1·1	0·8^c^	1·3	1^d^
Cholesterol (mg)	355·2	252	413·8	353·7^a^	349	213·5^b^	338·3	209·4^b^	319	183·9^b^
Fibre (g)	30·2	15·5	34·2	19·1^a^	31·1	15·2^b^	28·2	13·3^c^	27·1	12·7^c^

Differences were obtained by one-way ANOVA followed by Tukey’s *post hoc* test.

Different letters mean statistically significant differences (*P* ≤ 0·05).

Figure 1 shows the SC for the direct effects of the pathway analysis. The number of meals eaten each day (*P* < 0·001) and the sleep duration (*P* = 0·007) were negatively associated with UPF consumption, while the frequency of fried food intake (*P* < 0·001), the time spent on the computer (*P* = 0·031) and being employed (SC = 0·068; *P* = 0·024) were positively associated with UPF consumption. Being student did not have a significant direct effect on UPF consumption (SC = 0·051; *P* 0·084). The number of meals per d had a negative effect on the frequency of fried food intake (*P* < 0·001), whereas being employed had a positive effect on fried food intake weekly (SC = 0·098; *P* = 0·002). Students did not show a significant direct effect on fried food intake (SC = 0·053; *P* = 0·080). The time spent on the computer had a negative effect on the number of meals eaten each day (*P* = 0·048). None of the categories of the professional status showed association with the number of meals per d (employed: SC = 0·048; *P* = 0·112, and student: SC = 0·030; *P* = 0·313). The sleep duration had a positive effect from the time spent on the computer (*P* = 0·005) and a negative effect from the employed professional status (SC = 0·111; *P* ≤ 0·001). Being student was not associated with the sleep duration (SC = - 0·065; *P* = 0·069). Both professional status were associated with the time spent on the computer (employed: SC = 0·132; *P* ≤ 0·001, and student: SC = 0·147; *P* ≤ 0·001).

Table 3 presents, in addition to the direct effects, the indirect and total effects for the relationships of dietary, lifestyle and social variables with UPF consumption. Being employed was indirectly and positively associated with UPF consumption mediated by the sleep duration (*P* = 0·032) and fried food intake frequency (*P* 0·005). Being a student also had an indirect and positive association with UPF consumption mediated by time spent on the computer (*P* = 0·048). Finally, the number of meals eaten each day was negatively associated with UPF consumption mediated by the frequency of fried food intake (*P* < 0·001).

**Table 3:** Direct, indirect and total effects from path analysis

Relationship	Mediators	Effect	Standardized coefficient	*P*-value
Professional situation* – Employed → UPF consumption		Direct	0·068	0·024†
	Time on computer	Indirect	0·005	0·053
	Sleep		0·005	0·032†
	Meals/d		−0·005	0·125
	Fried food intake		0·011	0·005†
	Time on computer → Sleep		0·000	0·078
	Time on computer → Meals/d		0·001	0·086
	Sleep → Meals/d		0·000	0·521
	Time on computer → Fried food intake		0·000	0·543
	Sleep → Fried food intake		0·000	0·305
	Meals/d → Fried food intake		−0·001	0·140
	Time on computer → Sleep → Meals/d		0·000	0·529
	Time on computer → Sleep → Fried food intake		0·000	0·330
	Time on computer → Meals/d → Fried food intake		0·000	0·101
	Sleep → Meals/d → Fried food intake		0·000	0·523
	Time on computer → Sleep → Meals/d → Fried food intake		0·000	0·532
		Total	0·084	0·006†
Professional situation* – Student → UPF consumption		Direct	0·051	0·084
	Time on computer	Indirect	0·006	0·048†
	Sleep		0·003	0·133
	Meals/d		−0·003	0·320
	Fried food intake		0·006	0·094
	Time on computer → Sleep		0·000	0·073
	Time on computer → Meals/d		0·001	0·081
	Sleep → Meals/d		0·000	0·539
	Time on computer → Fried food intake		0·000	0·542
	Sleep → Fried food intake		0·000	0·356
	Meals/d → Fried food intake		0·000	0·328
	Time on computer → Sleep → Meals/d		0·000	0·528
	Time on computer → Sleep → Fried food intake		0·000	0·328
	Time on computer → Meals/d → Fried food intake		0·000	0·096
	Sleep → Meals/d → Fried food intake		0·000	0·541
	Time on computer → Sleep → Meals/d → Fried food intake		0·000	0·531
		Total	0·062	0·037†
Time on computer → UPF consumption		Direct	0·040	0·031†
	Sleep	Indirect	−0·003	0·053
	Meals/d		0·004	0·061
	Fried food intake		0·001	0·539
	Sleep → Meals/d		0·000	0·525
	Sleep → Fried food intake		0·000	0·318
	Meals/d → Fried food intake		0·000	0·076
	Sleep → Meals/d → Fried food intake		0·000	0·528
		Total	0·043	0·021†
Sleep → UPF consumption		Direct	−0·049	0·007†
	Meals/d	Indirect	0·001	0·514
	Fried food intake		−0·002	0·284
	Meals/d → Fried food intake		0·000	0·517
		Total	−0·050	0·007†
Meals/d → UPF consumption		Direct	−0·012	< 0·001†
	Fried food intake	Indirect	−0·012	< 0·001†
		Total	−0·124	< 0·001†
Fried food consumption		Direct	0·108	< 0·001†

* The professional status was categorised in dummy variables: employed; student; and retired or home duties or unemployed, and the retired or home duties or unemployed category was used as reference.

† Statistical significance (*P* < 0·05).

The final model was fully saturated (0 freedom degrees), which has a perfect fit. So, fit indices are not available.

## Discussion

Our data support the hypothesis that an accelerated pace of life due to the professional situation relates to sleep and time on the computer, and both predict dietary habits and have, as consequence, a higher UPF consumption among the CUME project’s participants.

This study points out the direct and indirect relationships between professional status and UPF consumption, even so employed individuals have less sleep duration and eat more frequently fried food, and both habits were associated with UPF consumption. The urban pace of life, with high working hours, demands foods that save consumers time and effort. That’s why an increasing trend in eating out in terms of frequency, the number of people and the percentage of total calories consumed is observed, besides the increase in the consumption of ready and semi-ready meals and easy-to-prepare foods^(18,39,40)^.

In this way, the choice of meals is influenced by cost, practicality and convenience. Time is a social determinant of health that is not frequently factored into the charge of food preparation, although it is a barrier to food preparation. In general, healthier home-cooked meals are cheaper than take-out meals or those eaten outside the home. However, home-made meals are 45 % more time demanding than other meals^(41)^. Thus, eating out is considered convenient as it does not involve cleaning and preparation time^(40)^. With less time available to prepare meals coupled with higher income, many households seem to increase their expenditure on out-of-home meals^(39)^. Eating out induces the substitution of traditional meals by snacks and fast food and is associated with unhealthy foods such as soda, sweets and fast food, all UPF^(40)^.

Students were more likely to spend more time on the computer, thus consuming more UPF in our study. Besides, students also are younger, and, therefore, are more exposed to nutritional transitions and UPF, which could shape their habits and food preference^(42–45)^. UPF are convenient, practical, and portable and can be consumed anywhere, including in front of the computer. On the other hand, cellphones, computers and television are environmental distractions that affect food consumption and decrease perceived satiety, thus increasing the quantity of UPF consumed^(1)^. Accordingly, eating in front of these devices should be avoided.

Our data showed a negative association between sleep duration and UPF consumption and the mediator role of sleep between being employed and UPF consumption. Both associations were expected, since working long hours has been related to reducing sleep duration in the current urbanised lifestyle^(25,26)^. The recommended sleep time for adults is 7 to 9 h every 24 h^(46)^. Sleeping periods lower than recommended should be analysed as risk behaviours associated with changes in appetite-regulating hormones^(25,27)^.

The number of meals eating per d and the high fried food intake were associated with the UPF consumption. The consumption of three main meals, including breakfast, combined with smaller meals, is described as part of a healthy diet capable of regulating metabolism and body weight^(29,30,47)^. An accelerated pace of life can damnify meal times, including the daily consumption of breakfast, supporting an energy overcompensation along the day^(28)^. Furthermore, eating more frequently during the day reduces hunger. It increases satiety, and appetite increases the desire to consume high-caloric foods, such as UPF^(29,30)^ and fried foods, which was a mediator between the number of meals eating per d and our outcome, the UPF consumption. Besides, the habit of consuming fried foods is considered unhealthy and is associated with the belief that healthy foods are tasteless^(47)^. UPF mainly contain fat, sodium and sugar. These additives intensify flavour, which may override endogenous satiety mechanisms and produce compulsion and addiction^(48)^.

Presently, there is an increasing trend in UPF consumption worldwide which is related to poor quality of diet^(10–12)^. Therefore, it is not surprising higher saturated fat intake in those subjects in the higher quartile, with a progressive increase in *trans*-fat intake, and a progressive decrease in fibre intake between the quartiles of UPF consumption.

There are connections between the current pandemic in NCD related to obesity and the recent changes in food production and distribution structures, mostly due to intrinsic characteristics of UPF that favour overconsumption: convenience, price and flavour^(20)^. This study collaborates with literature to elucidate the relationships between factors that shape food decisions on UPF consumption and points out that if the current globalised world results in an accelerated lifestyle, measures are necessary to reformulate industrialised foods in order to reduce the number of nutrients harmful to health. Besides that, nutritional education can promote autonomy among consumers allowing them to make better food choices.

### Limitations of the study

Although this is a cross-sectional analysis, the CUME project uses an online platform, which favours the recruitment of participants from different locations of Brazil and convenience regarding day and time to fill out the questionnaire^(31)^. Our participants represent a population with a high level of education, which guarantees reliable data since we depend on self-reported data^(31,34,49)^. Even if our food consumption data were obtained using an FFQ, these data were validated^(34)^, including concerning the NOVA classification, a scientifically recognised method to assess diet quality^(15)^. Although this study uses a high education sample, representativeness is usually not necessary in analytical epidemiological studies^(50)^. Thus, the CUME project provides data that allow the discussion, planning and implementation of specific interventions for Brazilians.

However, it is worth mentioning that others determinants that may also be associated with UPF consumption were not considered in this analysis, such as time and culinary skills^(1,4,18,20)^, food environment, different levels of income, education, and knowledge about health, food and nutrition^(21–23)^.

## Conclusion

Factors related to the current accelerated lifestyle, such as time spent on the computer, sleep duration and fried food consumption had a direct association with higher UPF consumption. These factors also acted as a mediator on the relationship between professional status and UPF consumption. Besides that, the number of meals eaten each day also was directly associated with UPF consumption. Our results indicate the complexity of unhealthy food choices and the need for multi-professional effort to inhibit them.
